# Long-term effects of SARS-CoV-2 infection in patients with and without chemosensory disorders at disease onset: a psychophysical and magnetic resonance imaging exploratory study

**DOI:** 10.1007/s10072-024-07429-4

**Published:** 2024-03-05

**Authors:** Maria Paola Cecchini, Francesca Benedetta Pizzini, Federico Boschi, Alessandro Marcon, Lucia Moro, Elizabeth Gordon, Nicolas Guizard, Enrica Cavedo, Maria Jimena Ricatti, Sheila Veronese, Stefano Tamburin, Michele Tinazzi, Giancarlo Mansueto, Andrea Sbarbati

**Affiliations:** 1 Department of Neurosciences, Biomedicines and Movement Sciences, Anatomy and Histology Section, Verona University, Strada Le Grazie 8, 37134 Verona, Italy; 2 Department of Engineering for Innovation Medicine, Radiology Unit, Verona University, Verona, Italy; 3 Department of Engineering for Innovation Medicine, Engineering and Physics Section, Verona University, Verona, Italy; 4 Department of Diagnostics and Public Heath, Unit of Epidemiology and Medical Statistics, Verona University, Verona, Italy; 5 Department of Infectious-Tropical Diseases and Microbiology, IRCCS Sacro Cuore, Negrar, Italy; 6 Qynapse, Paris, France; 7 Department of Neurosciences, Biomedicines and Movement Sciences, Neurology Unit, Verona University, Verona, Italy

**Keywords:** Post-COVID-19 patients, Smell/taste impairment, MRI brain volumetric analysis

## Abstract

A preserved sense of smell and taste allows us to understand many environmental “messages” and results in meaningfully improvements to quality of life. With the COVID-19 pandemic, it became clear how important these senses are for social and nutritional *status* and catapulted this *niche* chemosensory research area towards widespread interest. In the current exploratory work, we assessed two groups of post-COVID-19 patients who reported having had (Group 1) or not (Group 2) a smell/taste impairment at the disease onset. The aim was to compare them using validated smell and taste tests as well as with brain magnetic resonance imaging volumetric analysis. Normative data were used for smell scores comparison and a pool of healthy subjects, recruited before the pandemic, served as controls for taste scores. The majority of patients in both groups showed an olfactory impairment, which was more severe in Group 1 (median UPSIT scores: 24.5 Group 1 vs 31.0 Group 2, *p* = 0.008), particularly among women (*p* = 0.014). No significant differences emerged comparing taste scores between Group 1 and Group 2, but dysgeusia was only present in Group 1 patients. However, for taste scores, a significant difference was found between Group 1 and controls (*p* = 0.005). No MRI anatomical abnormalities emerged in any patients while brain volumetric analysis suggested a significant difference among groups for the right caudate nucleus (*p* = 0.028), although this was not retained following Benjamini–Hochberg correction. This exploratory study could add new information in COVID-19 chemosensory long-lasting impairment and address future investigations on the post-COVID-19 patients’ research.

**Supplementary Information:**

The online version contains supplementary material available at 10.1007/s10072-024-07429-4.

## Introduction

The coronavirus (SARS-CoV-2) disease, which emerged in late 2019 (COVID-19), became a global pandemic by March 2020 and associated with over 6 million deaths by 2023 (John Hopkins Coronavirus research center, https://coronavirus.jhu.edu/map.html). During the acute phase, various presentations occurred with symptoms ranging from common viral respiratory infection (e.g., fever, headaches, odynophagia, cough, and erythromelalgia) to severe dyspnea, pneumonia, and central nervous system disturbances, with an asymptomatic variant also reported. In addition, it became well known that many patients could also experience an olfactory and/or gustatory impairment [1–7].

The set of reported olfactory and gustatory deficits could be varied, including both quantitative (a reduction/loss of perception) and qualitative impairment (abnormal perception with or without stimulus). The mechanisms by which SARS-CoV-2 impairs chemosensory function are still under investigation, with molecular, histological, and imaging findings still limited and with no definitive conclusions [6, 8]. Regarding olfactory impairment, several hypotheses have been proposed. These include inflammatory damage of the olfactory mucosa and the axonal fibers as well as of the olfactory bulb, disruption of the olfactory neurons, their supporting cells, or injury to the neural pathways that convey chemosensory information to the brain [9–12]. Indeed, a major tropism of SARS-CoV-2 for the olfactory mucosa has been hypothesized [11, 13–15], even if a viral entry into the brain via olfactory mucosa seems unlikely [12].

Olfactory validated tests could discriminate between patients with COVID-19 and patients with acute cold, underlying the importance of a reliable olfactory evaluation. In particular, the identification performance assessment was reported to be able to discriminate the two clinical conditions, since the overall score was significantly lower in the COVID-19 patients compared to patients with acute cold [16, 17]. On the other hand, other studies not involving the common cold cases showed that the threshold performance was significantly more impaired for discrimination and identification abilities, suggesting that the virus might have less impact on performances for more cognitively demanding tasks such as the identification of smells [6]. Regarding the gustatory impairment, again studies suggested multiple mechanisms including the SARS-CoV-2 infection directly impacting the gustatory epithelial cells or the gustatory pathway or through indirect mechanisms such as inflammatory cytokine storm [7].

The reported recovery period from a COVID-19 chemosensory deficit generally occurs in a few weeks; however, it might persist much longer with substantial variability among patients [18]. This could have an impact on the quality of life, nutrition, mental health, and social interactions [19, 20]. To date, the current knowledge regarding the permanence of these chemosensory impairments is not yet clear [7].

In the current exploratory work, two groups of post-COVID-19 patients reporting having had and not having had a chemosensory impairment were evaluated several months following infection and the onset of these symptoms (Table 1). For both groups, we used validated tests for assessing olfactory and gustatory performances and these data were compared to volumetric measurements obtained by means of magnetic resonance imaging (MRI).

**Table 1:** Demographic, clinical, and chemosensory data of the COVID-19 patients

	Group 1	Group 2
Demographic data		
Number of participants	*n* = 20	*n* = 19
Age range (years)	42–66	40–67
Mean age ± SD (years)	55.8 ± 7.6	52.3 ± 7.9
Gender (M:F)	6:14	11:8
Days since COVID-19 onset median (Q1–Q3)	292 (246–362)	149 (136–219)
Hospitalization	*n* = 8	*n* = 8
Olfactory function		
Identification UPSIT mean ± SD	24.3 ± 8.0	30.5 ± 3.2
Identification UPSIT median (Q1-Q3)	24.5 (18.5–30.5)	31.0 (28.0–33.0)
Qualitative disorders: abnormal perception with or without stimulus (dysosmia)	*n* = 12	*n* = 1
Gustatory function		
Taste Strips Test (TST) score mean ± SD	10.6 ± 3.1	11.7 ± 2.7
Taste Strips Test (TST) score median (Q1–Q3)	11.0 (9.0–13.0)	12.0 (11.0–14.0)
Qualitative disorders: abnormal perception with or without stimulus (dysgeusia)	*n* = 18	*n* = 0

## Materials and methods

### Participants

An infectious disease specialist selected and recruited all research participants and classified them into two groups according to their chemosensory symptoms at the COVID-19 onset: Group 1 patients with smell/taste impairment and Group 2 without. Exclusion criteria were comorbidities affecting smell/taste performance (e.g., recent cranial traumatic event, otolaryngology disorders, recent stroke, and diabetes). All investigations were carried out in accordance with the Helsinki Declaration and its later amendments, and the protocol was approved by the Ethical Committee of the University Hospital of Verona (Prot. n. 6112, 01/02/2021). Written informed consent was obtained for each participant. Overall, 44 patients were initially recruited, 39 completing the study, following one individual withdrawing for personal reasons, one presented with MRI contraindications, and three could not complete the MRI session. Eight patients *per* group were hospitalized at the COVID-19 onset. All the other participants had an overall mild disease course that did not require hospitalization. All underwent detailed clinical neurological examination, smell, and taste evaluation as well as MRI investigation. Demographic and clinical features as well as olfactory/gustatory function assessment data of both groups are reported in Table 1.

In addition, a group of healthy subjects, previously evaluated for the gustatory function at Verona University, by means of the TST in the pre-COVID-19 period (2011 database), matched by age and gender with the COVID-19 patients (*n* = 37; F:19, M:18; age range 42–70 years; mean age 56.5 ± 7.9 years), was considered as a healthy control group for premorbid comparison only for the taste TST assessment.

### Olfactory and gustatory evaluation

#### University of Pennsylvania Smell Identification Test (UPSIT)

This test is one of the most widely used smell identification tests. It consists of four booklets, each containing ten “scratch and sniff” odorants, for 40 different odors. Each odorant is microencapsulated at the bottom of each booklets sheet. Odorous stimuli are released by scratching with a pencil tip in a standardized way. For each odor there are four written answer options and the participant is required to choose one of them in a forced choice manner. Every correct answer gives one point (maximum possible points = 40). Normosmia (normal olfactory function) is generally defined with a score in the range 34–40 for males and 35–40 for females. Microsmia (the reduction of perception) is subdivided in different classes (mild, moderate and severe) [21–23].

#### Taste strips test (TST)

This test is a detailed gustatory sensitivity test using 16 filter paper strips impregnated with the four taste qualities in four different concentrations (sweet: 0.4, 0.2, 0.1, 0.05 g/ml sucrose; sour: 0.3, 0.165, 0.09, 0.05 g/ml citric acid; salty: 0.25, 0.1, 0.04, 0.016 g/ml sodium chloride; bitter: 0.006, 0.0024, 0.0009, 0.0004 g/ml quinine hydrochloride). Participants place these filter paper strips on the middle of the tongue and after closing the mouth, the subject is asked to choose a taste quality from a list of four descriptors (sweet, sour, salty, and bitter). Before each strip administration, the subject should rinse the mouth with water. Each correct answer is given one point (maximum possible score is 16). A TST score ≥ 9 indicates normogeusia (normal gustatory function) while a TST score < 9 indicates hypogeusia. Patients may show ageusia (no taste perception) for certain taste qualities, while complete ageusia is rare and is diagnosed in patients with no sensation to the highest concentrations of all the four taste solutions. The test is quick and easy to administer. Normative values were obtained from over 500 individuals tested in a multicenter study [24, 25]. Taste strips qualities do not usually include umami taste (i.e., elicited by monosodium glutamate, some amino acids, and purine nucleotides), since this taste has been found to be poorly conceptualized by the European countries [25, 26].

### MRI assessment

The recruited participants underwent brain evaluation using a 3 T MRI scanner (Philips Elition S) equipped with a 32-channel array coil, located at the Radiology Department of the University Hospital of Verona. An experienced neuroradiologist, blinded to the chemosensory data of the patients, examined all MRI images. The MRI protocol included a standard morphological study protocol (3D, T1- and T2-weighted anatomical volumetric images) with the following parameters TR/TE = 8.1/3.7 ms, FOV = 240 × 240 mm^2^, 180 sagittal sections, 1 mm isotropic and TR/TE = 2200/249 ms, FOV = 240 × 240 mm^2^, 180 sagittal sections, 1 mm isotropic, respectively. These sequences made it possible to derive anatomical information with high spatial resolution and to exclude possible brain lesions/malformations. The 3D T1- and T2-weighted images were analyzed with QyScore® software (Qynapse, Paris, France) FDA-cleared and CE-marked neuroimaging analysis platform, approved for clinical use of automatic brain volumetry of cortical and deep white and grey matter quantification. Specifically, Qynapse provided the segmentation of all brain regions, including those involved in the olfactory circuit, i.e., piriform cortex as well as the olfactory tubercle and Broca’s olfactory cortex (Fig. S1). These segmented volumes were then compared to Qynapse large internal database of healthy normal controls and age and sex-matched population normed *z*-scores generated. For olfactory regions the automated anatomical labeling atlas 3 (AAL3) was considered [27]. In particular, a total of 44 brain areas were considered obtaining 42 *z*-score results and 2 AAL3-%ICV results of right and left olfactory cortex respectively. Volume %ICV is the volume of the left or right olfactory cortex expressed as a percentage of total intracranial volume (ICV) (see Table S1).

### Statistical analysis

Statistical analysis was performed by routines written in MATLAB R2018b software (MathWorks Inc., Natick, MA, USA) and using STATA software version 18.0 (StataCorp, College Station, Texas). Categorical variables were described with counts and percentages; symmetrical and asymmetrical quantitative variables were described using mean ± SD (standard deviation) or median with 1st and 3rd quartile (Q1-Q3), respectively. Normality was assessed using the Kolmogorov–Smirnov test. Since some variables had a non-normal distribution and due to the small sample size, non-parametric statistical analysis was adopted accordingly. To test differences between groups, Fisher’s exact test was used for categorical variables and Wilcoxon Mann–Whitney rank test or Kruskall-Wallis tests were used for quantitative variables. For smell, the UPSIT scores were compared to the 5th percentile of the UPSIT normative data matched for age and gender [23], and the difference were tested using the Wilcoxon matched-pairs signed-rank test. Differences were considered significant at *p* ≤ 0.05. Spearman’s coefficients were calculated to describe the correlation between every chemosensory evaluation variable and MRI volumetric values (*z*-score) for both groups, and exact *p*-values calculated both without and with Benjamini–Hochberg (B-H) correction for multiple testing, setting a False Discovery Rate (FDR) of 10%. The distribution of MRI *z*-score between COVID-19 groups was also assessed using Wilcoxon rank test and tested without and with B-H correction.

Furthermore, a statistical comparison was done among groups (Group 1, Group 2, and healthy control group) for the TST score results by means of the Wilcoxon Mann–Whitney test.

## Results

### Clinical neurological examination

During the neurological examination for Group 1 (with reported smell/taste impairment), no other post-COVID-19 symptoms were reported in the half of the recruited patients (10/20). Fatigue, mild memory difficulties, poor concentration, and insomnia were variously reported in the other half. For Group 2 (without reported smell/taste impairment), patients did not refer any other post-COVID-19 symptom, except one of them reporting mental fatigue, mild memory deficit and insomnia (1/19). None of the patients of Group 1 and Group 2 showed neurological deficit at the neurological physical examination.

### Olfaction

#### Olfactory function evaluation

No neurological disorders were reported or revealed during the clinical neurological examination. At olfactory assessment, almost all COVID-19 patients showed an olfactory impairment. In particular, the olfactory assessment for Group 1 showed one normosmia, five mild microsmia, three moderate microsmia, six severe microsmia, and five anosmia. Group 2 included two normosmia, nine mild microsmia, seven moderate microsmia, and one severe microsmia.

#### Comparison between COVID-19 groups

**Fig. 1:**
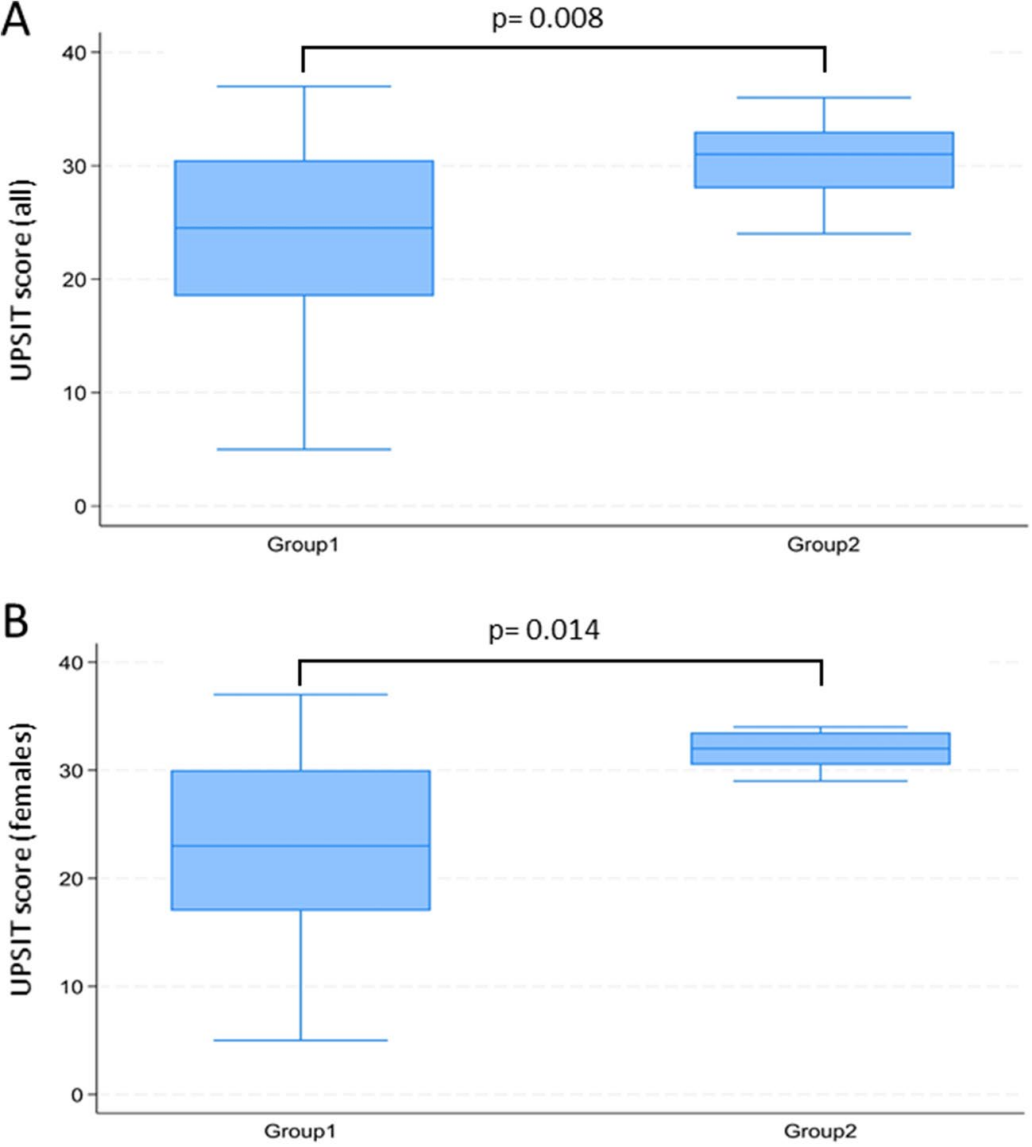
Comparison of UPSIT scores between COVID-19 patients in Group 1 and 2 (**A** males and females together; **B** females only). *p*-values from Wilcoxon Mann–Whitney rank tests

The UPSIT score was compared between the two groups (Table 1) and a significant difference emerged (median UPSIT scores: 24.5 Group 1 vs 31.0 Group 2, *p* = 0.008) also among female patients (23.0 Group 1 vs 32.0 Group 2, *p* = 0.014) (Fig. 1). In fact, the olfactory impairment was more severe in Group 1, based on UPSIT scores. No significant differences emerged between patients who were admitted to hospital during the acute infection and patients who were not (median UPSIT scores: 30 vs 29, *p* = 0.74). In addition, there were no differences between the two groups considering the qualitative disorder dysosmia.

#### Comparison with normative data

**Fig. 2:**
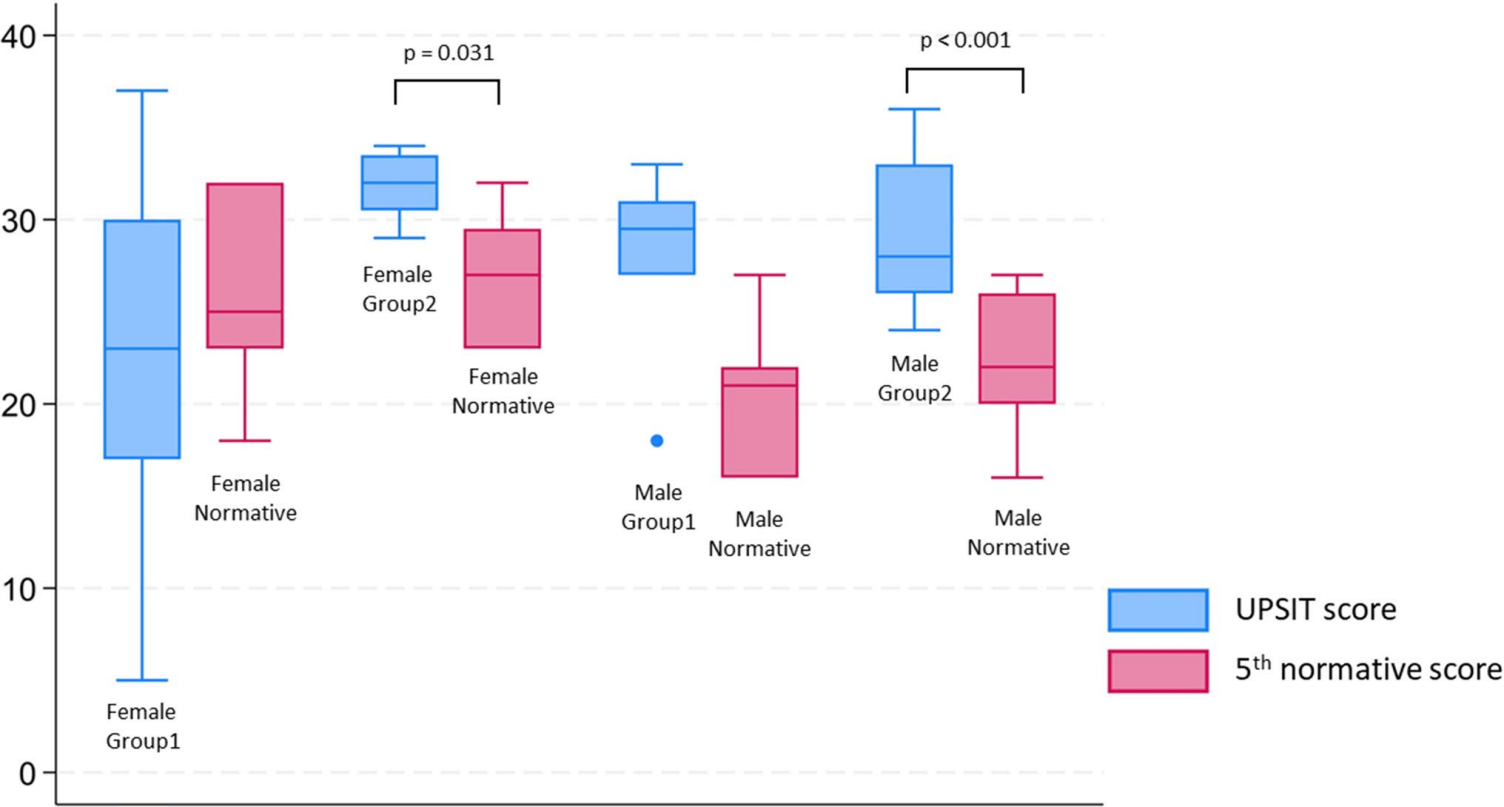
Comparison of UPSIT scores of COVID-19 patients from Group 1 and 2 with the 5th percentile of a normative population matched by sex. *p*-values from Wilcoxon matched-pairs signed-rank tests (*p*-values > 0.05 are not reported)

For both males (Figure S2) and females (Figure S3) the observed UPSIT scores were below the median score for the normative population: virtually all the patients had scores below the 25th percentile, corresponding to a degree of smell impairment from mild to severe. The impairment was the most severe among females belonging to Group 1, who showed a distribution of UPSIT scores equivalent to the bottom 5th percentile of the healthy age and sex-matched normative population (*p* = 0.246) (Fig. 2).

### Taste

#### Gustatory function evaluation

The gustatory assessment for Group 1 showed fifteen normogeusia, five hypogeusia (including two sweet, three sour and one bitter ageusia). Group 2 presented with seventeen cases of normogeusia, two of hypogeusia, and no single taste qualities ageusia cases.

#### Comparison between COVID-19 groups

The TST score was compared between the two groups (Table 1), and no significant differences emerged. Regarding qualitative disorders, dysgeusia could significantly distinguish between the two groups, being present only in Group 1 (*p* = 0.009) (Figure S4).

#### Comparison with a healthy sample

**Fig. 3:**
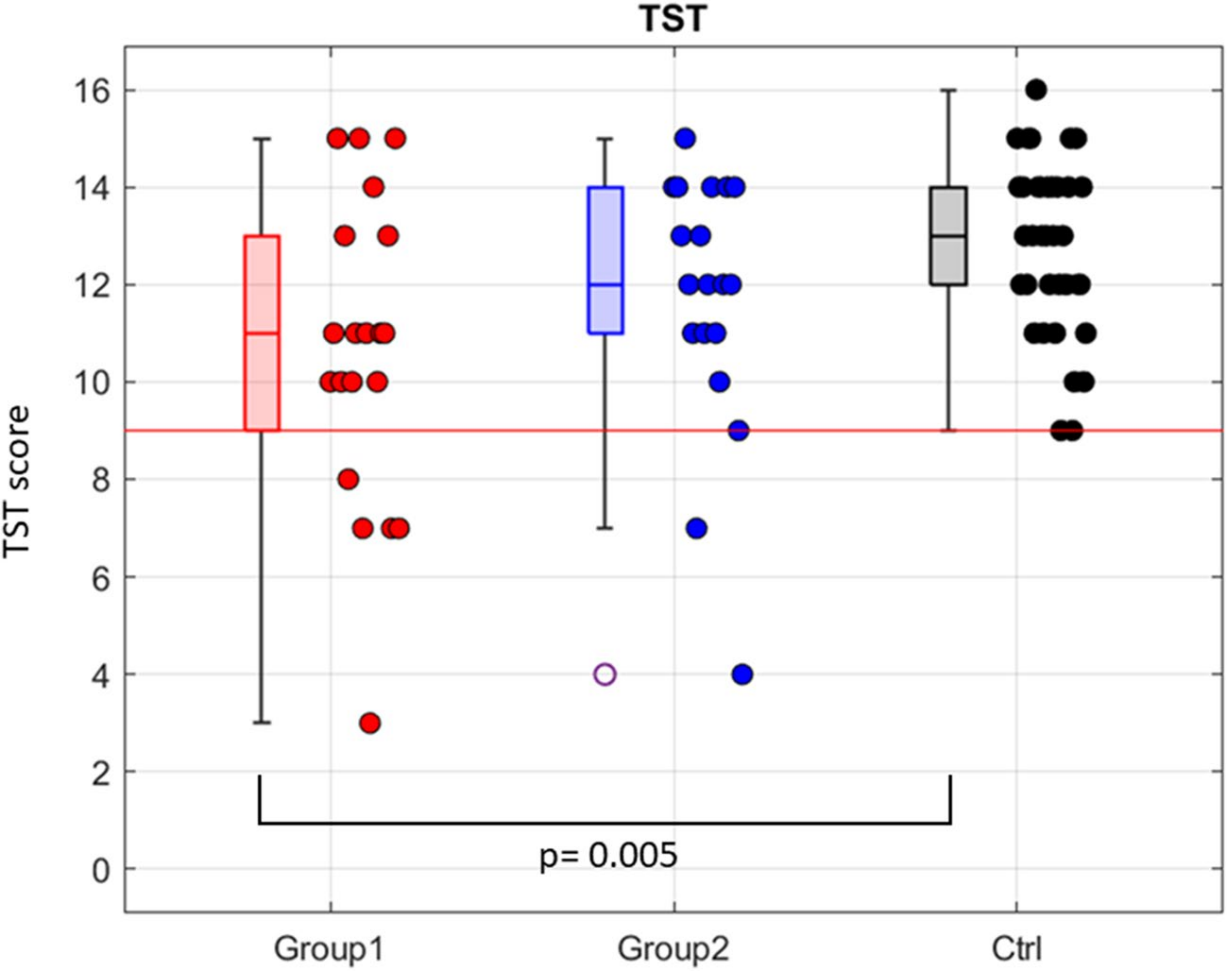
Comparison of the Taste Strips Test (TST) global score among groups: Group 1, Group 2 and the healthy control one (Ctrl). *p*-values from Wilcoxon matched-pairs signed-rank tests (*p-*values > 0.05 are not reported). A significant difference was found between Group 1 and Ctrl (*p* = 0.005). The normal range score is 9–16. The red line indicates the minimum normal score (9)

**Fig. 4:**
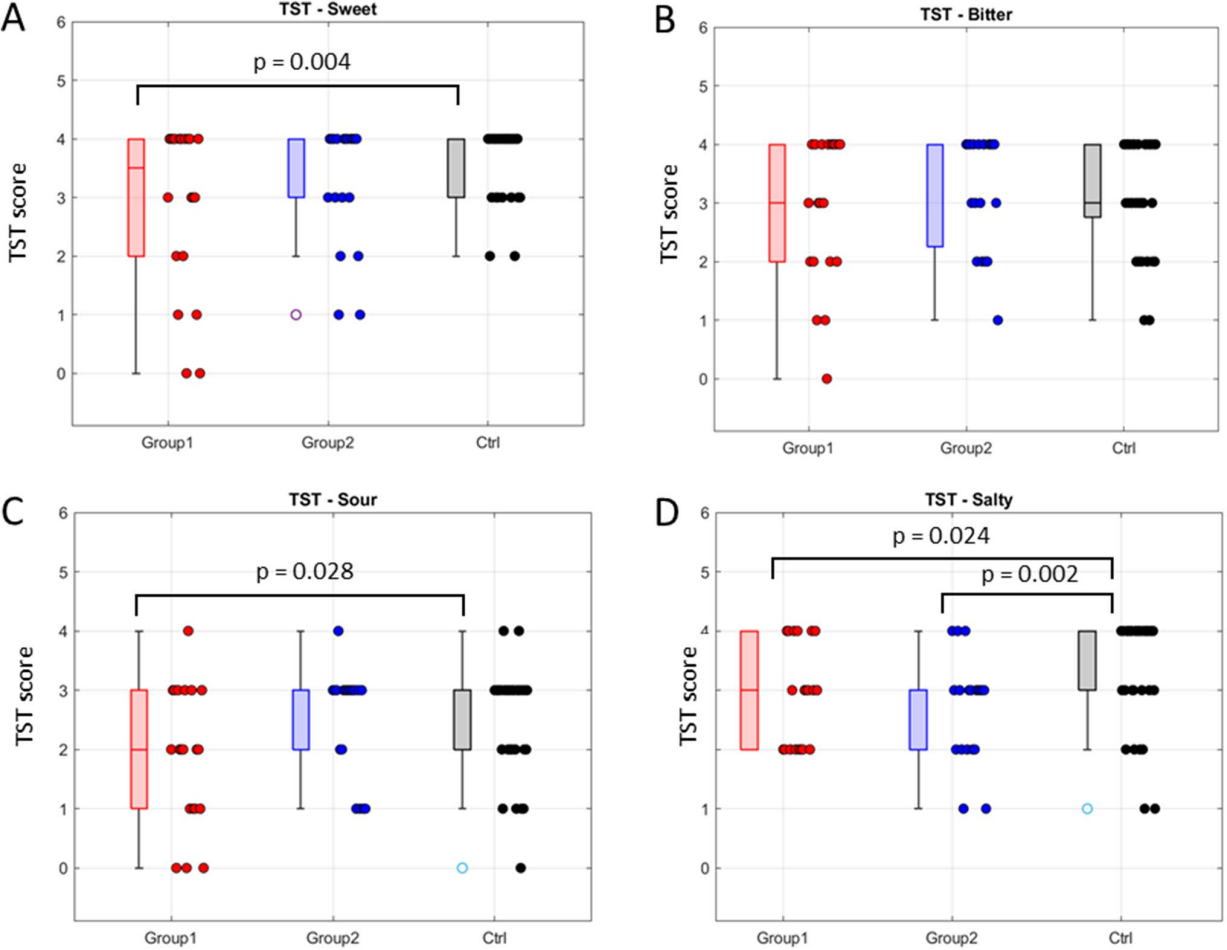
Comparison of the Taste Strips Test (TST) individual scores, for each taste quality assessed, among groups: Group 1, Group 2, and the healthy control one (Ctrl). Sweet (**A**), bitter (**B**), sour (**C**), and salty (**D**). *p*-values from Wilcoxon matched-pairs signed-rank tests (*p*-values > 0.05 are not reported). For sweet, sour, and salty, a significant difference emerged between Group 1 and Ctrl (*p* = 0.004, *p* = 0.028, and *p* = 0.024, respectively). In addition, for salty taste also Group 2 showed a significant difference with Ctrl (*p* = 0.002)

A significant difference was found between Group 1 and the control group for TST score (*p* = 0.005), as well as for sweet taste (*p* = 0.040) and sour taste (*p* = 0.028). For salty taste both groups were significantly different to the control group (Group 1 *p* = 0.024; Group 2 *p* = 0.002). For bitter taste, no significant difference was found between any group comparison (Figs. 3 and 4).

### MRI analysis

The preliminary radiological evaluation showed no evidence of clinically relevant brain abnormalities. The olfactory bulbs were clearly visible and showed a normal morphology. No sinunasal inflammatory processes or obstruction of the olfactory cleft were highlighted. MRI score values related to all the brain anatomical areas including the olfactory cortex were compared between the two groups and a significant difference was revealed for the right caudate nucleus (*p* = 0.028), being the medium *z*-score of − 0.35 ± 0.98 for Group 1 and 0.44 ± 0.99 for Group 2 (Table S1). No significant differences emerged, following B-H correction. In addition, after applying B-H correction there were no significant correlations among every chemosensory scores and MRI values for the two groups (Table S2).

## Discussion

In this exploratory study, we evaluated a pool of post-COVID-19 patients, several months following disease onset, who reported having had (Group 1) or not had (Group 2) chemosensory symptoms at the disease onset. All of them were assessed by means of validated smell and taste tests as well as through MRI brain volumetric analysis.

The results obtained highlight that the UPSIT identification score was significantly different between the two groups, with Group 1 demonstrating a greater olfactory deficit. Considering group and sex, a specific relation for females in Group 1 emerged, with an impairment equivalent to the bottom 5th percentile of the healthy female population (*p* = 0.246). No significant differences emerged between the two groups for the TST score.

The comparison with the healthy control group showed a significant difference, only with Group 1, for the TST score as well as for the sweet, sour, and salty tastes. As to qualitative disorders, dysgeusia significantly distinguishes between the two groups. Regarding MRI brain volumetric measures, a significant difference among groups for the right caudate nucleus (*p* = 0.028) emerged, even if no significant difference was retained following B-H correction as well as no significant correlation found between the MRI values and the chemosensory scores, following B-H correction.

Considering the olfactory *status*, when evaluated, several months after the disease onset, all the patients showed a smell identification deficit, except for three patients who were found to be normosmic (one in Group 1 and two in Group 2). Indeed, the smell identification deficit emerged in the majority of Group 2 patients, was mainly in the mild-moderate microsmia range, while in Group 1 this deficit was more severe (Figure S2, S3). Although all Group 2 patients reported no chemosensory impairment at the disease onset and at the time of the evaluation, this finding could point to a subclinical impact of the disease, detectable even several months after the COVID-19 onset and apparently without influence for the daily life. This result is in accordance with normative data comparison where the two groups showed a significant difference with respect to the median values of the normative sample (*p* = 0.020). Furthermore, despite the duration disease onset in Group 1 was longer than for Group 2, Group 1 still showed a more severe smell deficit, suggesting that the virus had a greater impact on the recovery process of these patients.

Regarding sex, an interesting finding is the particular relation between Group 1 olfactory deficit and being female. This result is in line with the meta-analysis of Tan and collaborators showing that the being female was strongly associated with lower odds of smell recovery in COVID-19; however, the underlying mechanism for this phenomenon is still not clear [28]. In fact, it is reported that males and females differ in their susceptibility and response to viral infections [29, 30] and hormonal reasons or immune related X chromosome linked genes may provide some insight into this result [31]. Previous research on the general post-viral olfactory impairment showed that females are more affected than males and that among women the vulnerable age range appears to be between 50 and 65 years, which is in line with the age general range of menopause or perimenopause. Indeed, in our exploratory work, our patients mean age falls within this range. Thus, it could be that the occurrence of SARS-CoV-2 infection during the perimenopause or menopause period makes females more vulnerable to the viral injury underlying persisting chemosensory deficit after several months, particularly for Group 1. In fact, this group represents the pool of patients with the referred chemosensory impairment at the onset and with a clear olfactory impairment at the psychophysical evaluation, several months after the onset.

When considering taste function, the TST assessment showed mainly normogeusia across both groups, and no significant difference was found between the two. Nevertheless, subjects with ageusia for some taste qualities were found in Group 1 (two sweet ageusia, three sour ageusia, and one bitter ageusia). When comparing the groups with the healthy control pool, a significant difference between Group 1 and controls emerged for the TST score as well as for the sweet, sour and salty taste scores. This is in line with previous reports showing single taste quality impairment, even of long duration [15, 16, 32]. This could highlight a taste peripheral injury, and among tastes, bitter seems to be more preserved and/or well recovered. Indeed, bitter taste is an important robust taste that warms against toxic and poisonous foods [33]. In addition, a salivary gland involvement with saliva secretory dysfunction might have a role also in the dysgeusia persistence [34]. This symptom, which was found to clearly distinguish the two groups and to persist several months after the onset, had also an important impact on the daily life quality, as referred by the patients. Indeed, the proteins commonly considered to be involved in the SARS-CoV-2 cellular entry (angiotensin-converting enzyme (2-ACE2) and the transmembrane serine protease (TMPRSS-2)), besides the olfactory epithelium, were found to be expressed also in the oral cavity including taste cells, gingival tissue and tongue’s epithelium as well as salivary glands [7, 35, 36].

No MRI anatomical abnormalities were revealed in all patients assessed. Looking at volumetric measures, a significant difference was revealed for the right caudate nucleus (*p* = 0.028) (Table S1). Nevertheless, following B-H correction, this difference was not retained, probably due to the small sample size. In addition, again after B-H correction, no significant correlation between MRI and chemosensory measures emerged, suggesting a more peripheral olfactory and gustatory system involvement. Despite these findings, the caudate nucleus could be considered an interesting site for future investigations, considering also its reported involvement in discrimination of odor quality and taste reward [37, 38]. However, because MRI was performed several months later the onset, we cannot rule out abnormalities in the acute phase [39]. In addition, other imaging techniques (e.g., fMRI, FDG-PET, and ASL-MRI) or brain connectivity studies might have revealed more information also on the functional processes of the chemosensory information, as these techniques have been shown to be sensitive to changes even in the presence of a normal brain MRI volumetric assessment [40–43]. Moreover, a more specific investigation on olfactory bulbs could have given further insights [44]. In addition, electroencephalographic (EEG) analysis, recently applied for evaluation of the post-COVID-19 cognitive symptoms [45], might be useful also to investigate the chemosensory sequelae, both qualitative and quantitative [15, 32], on large samples.

Summarizing, this is an exploratory study, focused on two different populations of post-COVID-19 patients, recruited for the presence/absence of a subjective impact on the chemosensory sphere at the onset and assessed only several months later. Our study has several limitations: we had no chemosensory and/or MRI data before COVID-19, which prevents us from assessing the exact impact of the disease on our findings and the patients sample size is small due to the long experimental session. Moreover, we could not recruit healthy COVID-19 free controls for MRI evaluation, because in that period a real non-COVID-19 pool of subjects would have been hard to find. Despite these limitations, it is significant to point out that all patients were well characterized and thoroughly investigated for smell and taste function also by means of validated tests. In most of all the patients, we found a smell impairment several months after the disease onset, showing a subclinical deficit also in Group 2 patients, and the referred dysgeusia could clearly distinguishing the two populations. This underlines the importance of combining validated smell/taste tests with a detailed clinical interview to have a more comprehensive picture of the chemosensory *status* of the patients (qualitative and quantitative). These data could add new insights into post-COVID-19 chemosensory impairment and inform future studies on large samples of patients presenting with documented smell/taste impairment persistence or not. This provides insights to help better understanding the features and the wide variability of the smell/taste recovery in this clinical condition.

### Supplementary Information

Below is the link to the electronic supplementary material.

Supplementary file1 (DOCX 410 KB)

## Data Availability

The data derived from this research are available on request to the corresponding author.
