# Supplementary material for: Long-term effects of SARS-CoV-2 infection in patients with and without chemosensory disorders at disease onset: a psychophysical and magnetic resonance imaging exploratory study
Source: Neurol Sci. 2024 Mar 5;45(6):2409–18. doi: 10.1007/s10072-024-07429-4 (PMC11082021; doi:10.1007/s10072-024-07429-4)
Supplement: Supplementary file 1 — Supplementary file1 (DOCX 410 KB) [file 10072_2024_7429_MOESM1_ESM.docx]

**Supplementary Information (SI)**


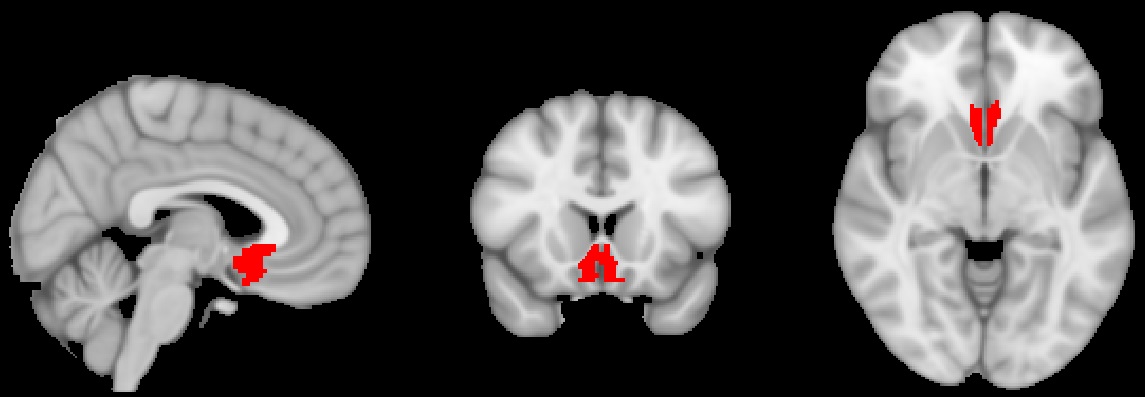


**Figure S1** Automated Anatomical Labeling Atlas 3 (AAL3) parcel of the olfactory cortex including the piriform cortex, as well as the olfactory tubercle, and Broca’s olfactory cortex.


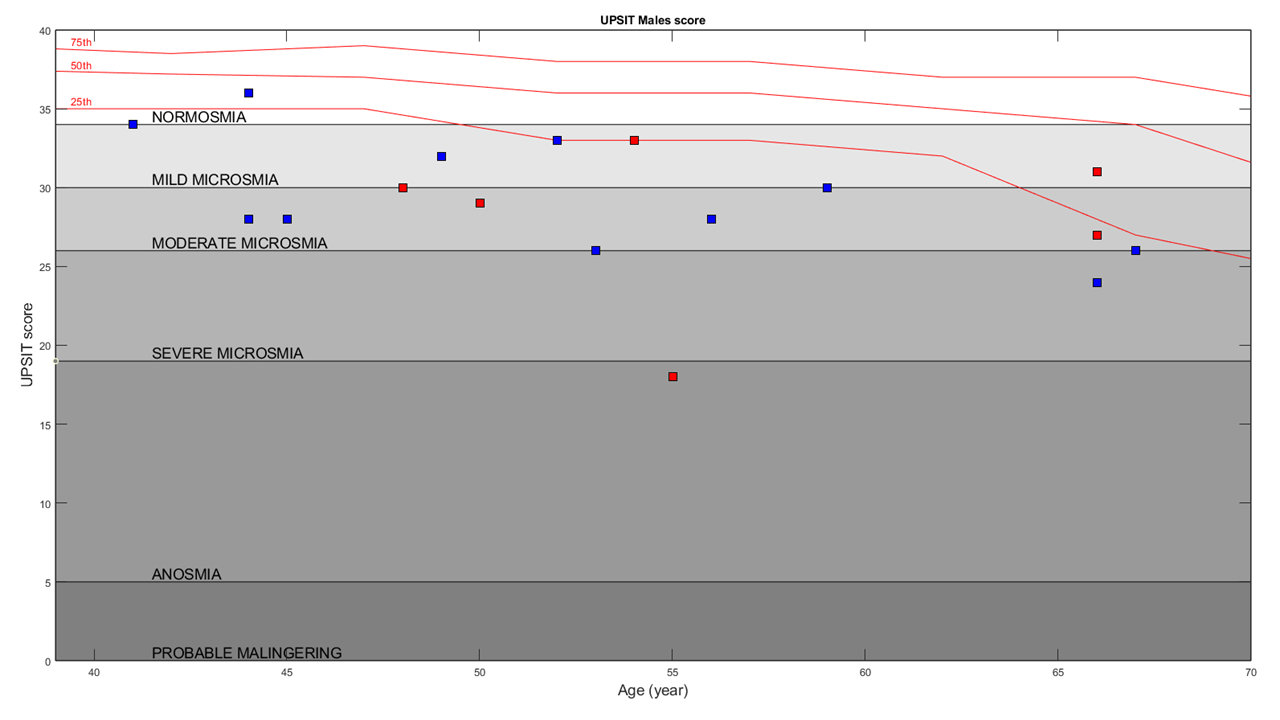


**Figure S2** UPSIT scores distribution for COVID-19 males (red squares are males of Group 1 and blue squares are males of Group 2). The majority of scores are below the 25th percentile mainly in the range of the mild-moderate olfactory microsmia (the three red lines represent the 25th, 50th and 75th percentile).


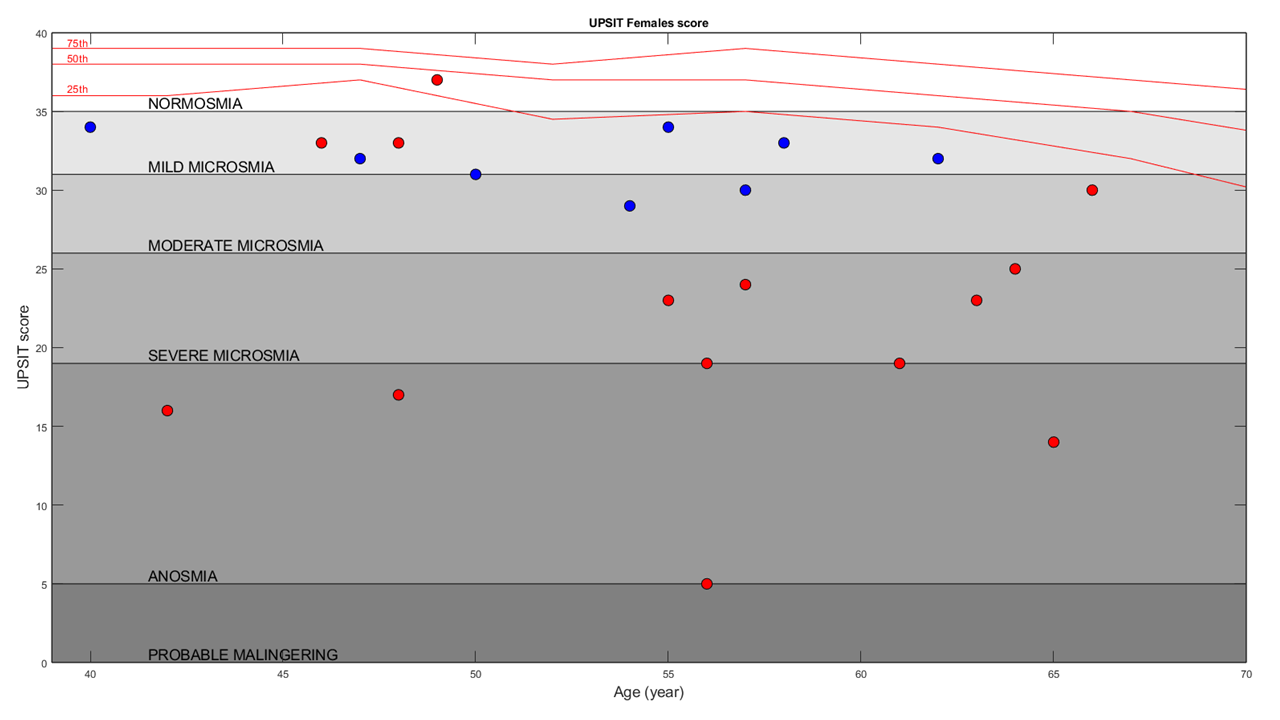


**Figure S3** UPSIT scores distribution for COVID-19 females (red dots are females of Group 1 and blue dots are males of Group 2). The majority of scores of the Group 1 are below the 25th percentile mainly in the range of the severe microsmia, while the Group 2 scores are in a less severe range of impairment (mild moderate microsmia) (the three red lines represent the 25th, 50th and 75th percentile).


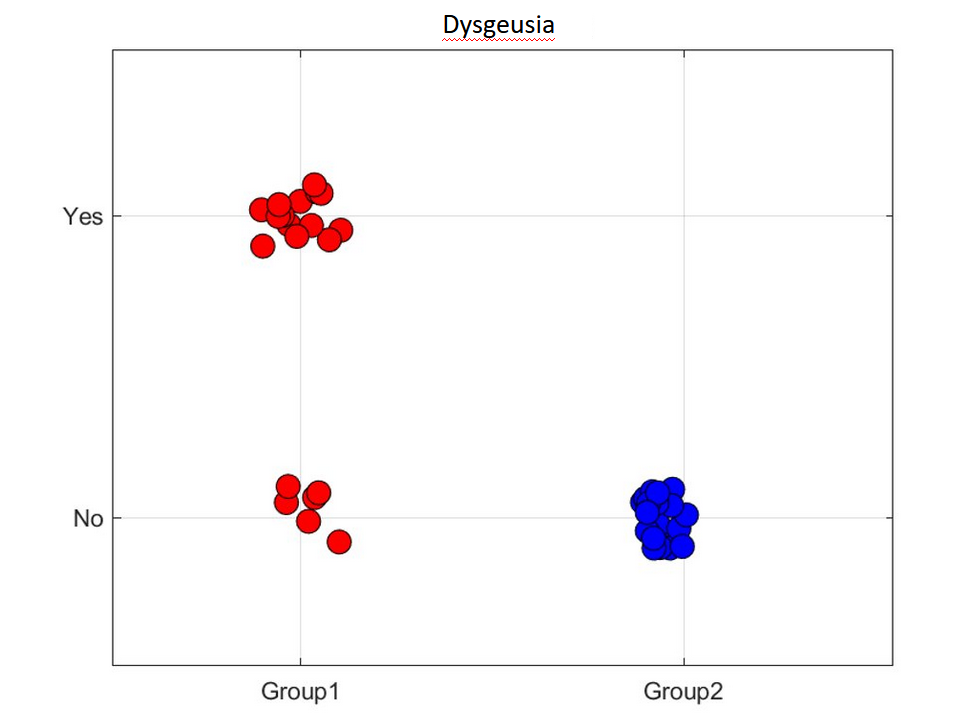


**Figure S4** Distribution of the qualitative gustatory disorders (dysgeusia) in the two groups of COVID-19 patients.

| **Anatomical brain areas** | ***p*-values** |
| --- | --- |
| right caudate nucleus (z-score) | **0.028** |
| caudate nucleus (z-score) | 0.061 |
| cortex of insula (z-score) | 0.065 |
| right globus pallidus (z-score) | 0.074 |
| globus pallidus (z-score) | 0.084 |
| cortex of left occipital lobe (z-score) | 0.084 |
| cortex of temporal lobe (z-score) | 0.084 |
| cortex of right temporal lobe (z-score) | 0.084 |
| right amygdala (z-score) | 0.089 |
| putamen (z-score) | 0.089 |
| right putamen (z-score) | 0.089 |
| cortex of left insula (z-score) | 0.095 |
| cortex of right insula (z-score) | 0.095 |
| right hippocampus (z-score) | 0.10 |
| left thalamus (z-score) | 0.10 |
| amygdala (z-score) | 0.11 |
| cortical gray matter (z-score) | 0.11 |
| cortex of occipital lobe (z-score) | 0.11 |
| cortex of left temporal lobe (z-score) | 0.12 |
| cortex of right frontal lobe (z-score) | 0.13 |
| left putamen (z-score) | 0.13 |
| thalamus (z-score) | 0.13 |
| hippocampus (z-score) | 0.14 |
| cortex of left frontal lobe (z-score) | 0.15 |
| left globus pallidus (z-score) | 0.16 |
| brainstem (z-score) | 0.17 |
| cortex of right occipital lobe (z-score) | 0.17 |
| cortex of frontal lobe (z-score) | 0.17 |
| cortex of parietal lobe (z-score) | 0.19 |
| left hippocampus (z-score) | 0.20 |
| left caudate nucleus (z-score) | 0.21 |
| cortex of left limbic lobe (z-score) | 0.22 |
| cortex of left parietal lobe (z-score) | 0.22 |
| right thalamus (z-score) | 0.25 |
| AAL3_right Olfactory cortex_volume_%ICV* | 0.25 |
| cortex of right parietal lobe (z-score) | 0.26 |
| cerebellum (z-score) | 0.27 |
| cortex of limbic lobe (z-score) | 0.35 |
| left amygdala (z-score) | 0.44 |
| cortex of right limbic lobe (z-score) | 0.44 |
| gray matter of cerebellum (z-score) | 0.50 |
| AAL3_left Olfactory cortex_volume_(%ICV)* | 0.50 |
| white matter (z-score) | 0.65 |
| lateral ventricle (z-score) | 0.73 |

**Table S1** *p*-values for the comparison of MRI brain areas between the two groups from Wilkoxon Mann-Whitney rank tests with no adjustment for multiple testing. A significant difference was revealed for the right caudate nucleus (*p*=0.028), being the medium z-score -0.35 ± 0.98 for the Group 1 and 0.44 ± 0.99 for the Group 2. No significant differences emerged following Benjamini-Hochberg correction. *volume %ICV is the volume of the left or right Olfactory cortex expressed as a percentage of total intracranial volume (ICV)

| **Spearman’s correlations (UPSIT smell score and MRI areas). *p*-value range** | | | | | |
| --- | --- | --- | --- | --- | --- |
| **Group 1** | **Group 2** | **Group 1 + Group 2** | **Group 1**  **B-H correction** | **Group 2**  **B-H correction** | **Group 1 + Group 2**  **B-H correction** |
| **min 0.044**  max 0.882 | min 0.149  max 0.994 | **min 0.027**  max 0.886 | min 0.501  max 0.898 | min 0.999  max 1.012 | min 0.238  max 0.886 |
|  | | | | | |
| **Spearman’s correlations (TST taste score and MRI areas). *p*-value range** | | | | | |
| **Group 1** | **Group 2** | **Group 1 + Group 2** | **Group 1**  **BH correction** | **Group 2**  **B-H correction** | **Group 1 + Group 2**  **B-H correction** |
| min 0.052*  max 0.972 | **min 0.037**  max 0.994 | min 0.057  max 0.996 | min 0.481  max 0.972 | min 0.344  max 1.012 | min 0.474  max 0.996 |

**Table S2** *p*-values range (min-max) for Spearman’s rank correlations of UPSIT smell test and TST taste test with MRI brain volumetric measures (z-scores) (null hypothesis: no correlation). *p*-values are reported for Group 1 and 2 separately and together, with and without Benjamini-Hochberg (B-H) correction. Without corrections, for the UPSIT score and Group 1 a significant correlation emerged with MRI volume of left parietal lobe cortex (*p*=0.044). Considering the two groups together, a significant correlation emerged with the MRI volume of occipital lobe cortex (*p*=0.038), left occipital lobe cortex (*p*=0.027), right occipital lobe cortex (*p*=0.049), parietal lobe cortex (*p*=0.043), left parietal lobe cortex (*p*=0.040) and temporal lobe cortex (*p*=0.035). Without corrections, there was a significant correlation with MRI volume of the left amygdala cortex (*p*=0.043) and white matter (*p*=0.037) for TST score and Group 2. In addition, again for TST score, a close to be significant correlation was found for Group 1 with right caudate nucleus (*p*=0.052*). No significant differences emerged following B-H correction.
